# Clonal hematopoiesis of indeterminate potential and cardiovascular disease: mechanistic insights, clinical implications, and the dawn of precision cardio-hematology

**DOI:** 10.3389/fcvm.2026.1796328

**Published:** 2026-04-10

**Authors:** Hong Wu, Xinyi Meng, Long Li, Chao Zhang

**Affiliations:** Department of Cardiovascular Medicine, The People's Hospital of Pingyi County, Shandong, China

**Keywords:** atherosclerosis, CHIP, clonal hematopoiesis of indeterminate potential, DNMT3A, heart failure, inflammation, NLRP3 inflammasome, precision medicine

## Abstract

Clonal hematopoiesis of indeterminate potential (CHIP) has substantially advanced our understanding of cardiovascular pathogenesis by demonstrating how somatic mutations in hematopoietic stem cells amplify the bone marrow–heart axis to accelerate atherosclerosis and heart failure, building upon the established role of hematopoietic cells in vascular inflammation. Characterized by the age-associated expansion of hematopoietic stem cell clones harboring somatic mutations—most commonly in TET2, DNMT3A, ASXL1, and JAK2—CHIP affects over 10% of individuals older than 70 years and confers a 1.5- to 2-fold increased risk of coronary heart disease and all-cause mortality, independent of traditional cardiovascular risk factors. This review synthesizes current evidence on the epidemiological associations, molecular mechanisms, and therapeutic implications of CHIP in cardiovascular disease. We critically examine how loss-of-function mutations in epigenetic regulators promote a pro-inflammatory macrophage phenotype through NLRP3 inflammasome activation and IL-1β/IL-6 signaling, thereby accelerating atherogenesis, plaque instability, and myocardial fibrosis. Notably, the cardiovascular impact of CHIP exhibits marked gene-specific heterogeneity: TET2 mutations confer the highest risk and demonstrate the greatest responsiveness to anti-inflammatory therapies, whereas DNMT3A mutations show more modest associations and may operate through distinct pathways. The CANTOS trial exploratory analyses and subsequent studies suggest that IL-1β inhibition with canakinumab and colchicine may preferentially benefit TET2-mutant CHIP carriers, suggesting a future of mutation-guided cardiovascular prevention. However, significant knowledge gaps remain, including the lack of prospective, genotype-stratified clinical trials and limited data in non-European populations. We propose that CHIP represents not merely a risk marker but a modifiable therapeutic target, and advocate for the emergence of “Cardio-Hematology” as a new subspecialty bridging hematology and cardiovascular medicine. As the field evolves from discovery to translation, precision approaches targeting specific CHIP genotypes may fundamentally transform cardiovascular risk stratification and treatment.

## 1 Introduction

### 1.1 Redefining cardiovascular risk: beyond traditional paradigms

Despite remarkable advances in the treatment of hypertension, diabetes, and dyslipidemia, cardiovascular disease (CVD) remains the leading cause of mortality worldwide. A sobering reality confronts clinicians daily: traditional risk factors—the cornerstones of preventive cardiology for half a century—explain only approximately 50% of incident cardiovascular events (1). This “residual risk” has long puzzled the cardiology community, driving the search for novel pathogenic mechanisms and therapeutic targets.

The inflammatory hypothesis of atherosclerosis has gained substantial validation through clinical trials demonstrating that IL-1β inhibition reduces cardiovascular events independent of lipid lowering. Yet this conceptual breakthrough raised a fundamental question: what drives this pathological inflammation in the first place? The discovery of clonal hematopoiesis of indeterminate potential (CHIP) provides a compelling answer, revealing that the bone marrow—traditionally the domain of hematologists—plays a previously unrecognized role in cardiovascular pathogenesis.

Importantly, the fundamental contribution of bone marrow-derived hematopoietic cells to atherosclerosis was well established before the discovery of CHIP. Pioneering studies by Tall, Yvan-Charvet, and colleagues demonstrated that cholesterol efflux pathways in hematopoietic stem cells are central regulators of myeloproliferation and vascular inflammation (2), while Swirski, Nahrendorf, and others established the hematopoietic contribution to myocardial inflammation and repair. The emergence of CHIP advances this established “bone marrow–heart axis” into a new dimension: somatic mutations in hematopoietic stem cells amplify these inflammatory processes to accelerate cardiovascular disease (1, 3). This conceptual evolution marks cardiovascular medicine's entry into the “somatic mutation era”—an era where acquired genetic alterations in non-cardiac tissues profoundly influence cardiac outcomes.

### 1.2 CHIP: definition and historical context

CHIP is defined as the presence of somatic mutations in hematopoietic cells at a variant allele fraction (VAF) ≥2%, in the absence of overt hematologic malignancy or unexplained cytopenias (4). The condition arises from the clonal expansion of hematopoietic stem cells (HSCs) harboring mutations that confer a selective proliferative advantage. The most frequently mutated genes are the epigenetic regulators TET2 and DNMT3A, followed by ASXL1, JAK2, TP53, and splicing factors such as SF3B1 and SRSF2 (1, 3).

The recognition of CHIP's cardiovascular significance emerged through large-scale exome sequencing studies, which identified age-associated clonal hematopoiesis and, unexpectedly, found strong associations with cardiovascular mortality rather than hematologic malignancy. This observation—that mutations traditionally associated with leukemia predisposition were more commonly killing patients through heart attacks and strokes—fundamentally reshaped our understanding of the condition's clinical relevance (1, 3, 5).

Subsequent mechanistic work established biological plausibility. In 2017, Fuster and colleagues demonstrated that Tet2-deficient hematopoietic cells accelerate atherosclerosis in murine models through NLRP3 inflammasome-mediated IL-1β secretion (6), and in the same year Jaiswal et al. established in a major prospective human cohort study that CHIP carriers face a roughly twofold higher risk of coronary heart disease and early-onset myocardial infarction (7). This study moved CHIP from an epidemiological association to a mechanistically grounded cardiovascular risk factor. Sano et al. further demonstrated that TET2-mediated clonal hematopoiesis accelerates heart failure through the IL-1β/NLRP3 inflammasome pathway (8).

Beyond atherosclerosis and heart failure, growing evidence implicates CHIP in a broader spectrum of cardiovascular disorders. Epidemiological studies have linked CHIP to an increased risk of stroke (9–11), cardiac arrhythmias including atrial fibrillation (12, 13), myocarditis and pericarditis (14), peripheral artery disease (15), and aortopathy (16). Additionally, JAK2-CHIP mutations are strongly associated with venous and arterial thrombosis. This expanding spectrum reflects the systemic nature of CHIP-driven inflammation and its broad impact across the cardiovascular system.

### 1.3 Scope and perspective of this review

This review aims to provide more than a systematic compilation of the literature. As clinicians confronting CVD daily, we offer a critical appraisal of the evidence, highlighting not only what is known but also what remains uncertain. We address several key questions that are paramount for translating CHIP research into clinical practice:

- Is CHIP merely a biomarker of biological aging, or a causal driver of cardiovascular disease?
- Can CHIP be therapeutically targeted, and if so, which patients would benefit most?
- How should the practicing cardiologist incorporate CHIP into clinical decision-making?

We argue that CHIP offers a concrete basis for precision cardiovascular medicine—an approach stratified not by traditional phenotypes but by underlying molecular genotypes. The journey from discovery to clinical translation is underway, and this review charts the current landscape while anticipating the road ahead. While no official clinical screening guidelines for CHIP currently exist, the American Heart Association has recently published a Scientific Statement on Clonal Hematopoiesis and Its Cardiovascular Implications (17), representing an important first step toward consensus-based clinical guidance.

## 2 Epidemiology: CHIP and atherosclerotic cardiovascular disease

### 2.1 Strength and consistency of epidemiological evidence

The association between CHIP and atherosclerotic CVD has been replicated across multiple large cohorts, meta-analyses, and diverse populations, establishing CHIP as a robust independent risk factor. Individuals with CHIP demonstrate a 1.5- to 2-fold increased risk of coronary heart disease (CHD) and a 1.2- to 1.4-fold increased risk of all-cause mortality after adjustment for traditional cardiovascular risk factors including age, sex, hypertension, diabetes, hyperlipidemia, and smoking status (1, 3, 18, 19).

The magnitude of this risk increase is clinically meaningful and warrants careful consideration. To contextualize, the hazard ratio conferred by CHIP for incident CHD (approximately 1.5–2.0) is comparable to that of diabetes mellitus and exceeds that of many traditional risk factors in certain populations (5). The excess cardiovascular—rather than hematologic—mortality burden of CHIP is substantial; the annual risk of transformation to overt hematologic malignancy is only 0.5%–1%, whereas the cardiovascular mortality burden is substantially higher (1, 3). Not all studies have confirmed this association, however: the ASPREE cohort, examining CHIP in healthy community-dwelling elderly individuals, did not demonstrate a significant association with incident cardiovascular events (20), highlighting that CHIP's cardiovascular impact may be attenuated in populations free of established atherosclerosis at baseline. This finding should be considered when interpreting population-level risk estimates and may be relevant to therapeutic decision-making in older adults, particularly in the context discussed in Section 2.3.

Meta-analytic data have strengthened these observations. Singh et al. conducted a systematic review and meta-analysis encompassing multiple studies and confirmed consistent associations between CHIP and adverse cardiovascular outcomes, with evidence quality rated as moderate to high (19). The consistency across heterogeneous study designs—prospective cohorts, case-control studies, and biobank analyses—supports a genuine biological relationship rather than methodological artifact.

From a clinical standpoint, these data compel us to reconsider CHIP as a cardiovascular risk factor on par with traditional variables. The magnitude of risk is not trivial; a 50%–100% increase in CHD risk demands attention equivalent to that given to hypertension or dyslipidemia.

### 2.2 Heterogeneity of risk: not all CHIP is created equal

A critical insight emerging from recent research—and one that has profound implications for clinical practice—is that CHIP should not be conceptualized as a monolithic entity. The cardiovascular risk associated with CHIP varies substantially according to the specific mutated gene, clone size, and clinical context (1, 3, 18, 19).

**Clone size matters.** The variant allele fraction (VAF) demonstrates a dose-response relationship with cardiovascular outcomes. Individuals with larger clones (VAF >10%) face significantly higher risks than those with smaller clones (VAF 2%–10%), suggesting that the burden of mutant cells directly influences pathogenic potential (21, 22). This observation has important clinical implications: not all CHIP-positive individuals warrant equal concern. A patient with a small TET2 clone (VAF 3%) carries meaningfully different risk than one with a large clone (VAF 15%).

**Gene-specific risk stratification is essential.** Among the major CHIP driver genes, TET2 and JAK2 mutations consistently confer the highest cardiovascular risk, with hazard ratios often exceeding 2.0 for incident CHD (18, 23). In contrast, DNMT3A mutations—despite being the most prevalent—demonstrate more modest and sometimes inconsistent associations with cardiovascular outcomes (19, 24). This heterogeneity reflects fundamental differences in the downstream pathogenic mechanisms, as we will discuss in subsequent sections.

From a clinical standpoint, this heterogeneity mandates a nuanced approach that our field has been slow to adopt. Reporting a patient as “CHIP-positive” without specifying the mutated gene and clone size is analogous to diagnosing “cancer” without identifying the organ of origin—technically accurate but clinically insufficient. We advocate for genotype-specific risk communication and, ultimately, genotype-specific management strategies.

### 2.3 CHIP in special populations: context matters

The cardiovascular impact of CHIP varies across clinical contexts, highlighting the importance of population-specific considerations that clinicians must appreciate.

**Established coronary artery disease.** Several studies have examined CHIP in patients with known CAD, yielding mixed results that merit careful interpretation. Gumuser et al. demonstrated that CHIP predicts adverse outcomes even in patients with established atherosclerotic CVD, suggesting additive prognostic value beyond traditional risk stratification (18). Most recently, Von Scheidt et al. confirmed in a large-scale cohort study that CHIP is independently associated with increased mortality in patients with coronary artery disease (25), providing further evidence of the prognostic importance of clonal hematopoiesis in this population. However, other studies in highly selected populations—such as patients undergoing coronary angiography or heart transplant recipients—have reported attenuated or null associations (20, 26, 27). These discrepancies likely reflect competing risks, survivor bias, and differences in background cardiovascular risk. The apparent attenuation in high-risk populations may represent a ceiling effect rather than absence of biological impact.

**Advanced age.** A particularly intriguing observation is that the relative risk conferred by CHIP appears to attenuate in the very elderly (28, 29). In individuals over 80 years, CHIP prevalence exceeds 20%, yet its incremental prognostic value may diminish. Whether this reflects biological saturation of inflammatory pathways, competing mortality from other causes, or methodological limitations remains uncertain. This observation should not, however, lead to therapeutic nihilism in older patients with CHIP. The ASPREE cohort—which examined CHIP in healthy community-dwelling elderly individuals—did not demonstrate a significant association between CHIP and incident cardiovascular events (22), providing additional support for the concept that CHIP's cardiovascular impact may be attenuated in populations free of established atherosclerosis. Taken together, these findings suggest that biological context, comorbidity burden, and baseline cardiovascular risk substantially modify CHIP's incremental prognostic contribution in older adults.

**Non-European populations**. A critical limitation of the current evidence base—and one that raises important equity concerns—is its predominant derivation from European-ancestry cohorts. Data on CHIP prevalence, mutation spectrum, and cardiovascular associations in African, Asian, and Hispanic populations remain sparse (1). Given potential differences in genetic background, environmental exposures, and healthcare access, addressing this disparity is essential for the equitable translation of CHIP-targeted therapies.

## 3 CHIP and heart failure: a tale of two phenotypes

CHIP has emerged as an independent risk factor for incident heart failure and an adverse prognostic marker in established disease. A large prospective population-based cohort study from the UK Biobank, encompassing 417,616 participants—providing adequate statistical power for gene-specific analyses of less frequent CHIP mutations—recently demonstrated that CHIP was significantly associated with incident heart failure, with TET2- and DNMT3A-CHIP conferring the highest risks (30). Complementary meta-analyses have further confirmed elevated rates of heart failure hospitalization and cardiovascular mortality in CHIP carriers compared with non-carriers (22, 29). These findings establish CHIP as a modifiable upstream contributor to heart failure in the general population and highlight the importance of distinguishing its impact across the two major heart failure phenotypes.

### 3.1 CHIP in heart failure with reduced ejection fraction

The relationship between CHIP and heart failure with reduced ejection fraction (HFrEF) has been explored in several cohort studies, with consistent findings that have important clinical implications. CHIP, particularly involving TET2 and DNMT3A mutations, is associated with increased incidence of HFrEF and worse prognosis among those with established disease (31, 32). Mechanistically, CHIP-driven inflammation may accelerate adverse cardiac remodeling, promote myocardial fibrosis, and impair contractile function through sustained IL-1β and IL-6 signaling.

Assmus et al. demonstrated that clone size provides prognostic information in chronic ischemic heart failure, with larger DNMT3A and TET2 clones predicting higher mortality and heart failure hospitalization rates (21). These findings suggest that CHIP monitoring could enhance risk stratification beyond conventional biomarkers such as natriuretic peptides—an intriguing possibility that warrants prospective validation.

In clinical practice, the association between CHIP and HFrEF progression suggests that anti-inflammatory strategies might benefit a subset of heart failure patients, particularly those with documented TET2 mutations. However, this hypothesis remains to be tested in dedicated clinical trials.

### 3.2 HFpEF: the ideal stage for CHIP pathobiology

Heart failure with preserved ejection fraction (HFpEF) represents a particularly compelling context for CHIP research, and arguably offers the most promising therapeutic opportunity. HFpEF is increasingly recognized as a systemic, inflammation-driven syndrome rather than a purely cardiac disorder (33). Its pathophysiology involves microvascular inflammation, endothelial dysfunction, and myocardial fibrosis—processes that align closely with the inflammatory phenotype promoted by CHIP.

Recent evidence strongly implicates CHIP in HFpEF pathogenesis. Cochran et al. demonstrated that TET2 mutations are enriched in HFpEF patients and that Tet2-deficient mice develop diastolic dysfunction and cardiac fibrosis (34). Schuermans et al. confirmed these associations in a large population-based cohort, showing that CHIP—particularly TET2-CHIP—independently predicts incident HFpEF after adjustment for traditional risk factors (35). Pandey et al. provided crucial mechanistic insight, demonstrating that TET2-mutant clonal hematopoiesis promotes HFpEF through NLRP3 inflammasome-driven cardiac macrophage activation and fibrosis; genetic deletion of NLRP3 attenuated the HFpEF phenotype (36).

From a clinical perspective, these findings have direct clinical relevance. HFpEF has long been a therapeutic wasteland, with most trials of conventional heart failure therapies yielding neutral results. The lack of effective therapies has been one of the most frustrating challenges in contemporary cardiology. The identification of CHIP as a driver of HFpEF pathophysiology opens new therapeutic avenues—specifically, anti-inflammatory strategies targeting the NLRP3-IL-1β axis may benefit a genetically defined subset of HFpEF patients.

### 3.3 Clinical implications: toward molecular phenotyping of heart failure

The differential impact of CHIP across heart failure subtypes points to an unmet need for molecular phenotyping in heart failure management, an approach our field must increasingly adopt. We propose that CHIP status, combined with mutation-specific information and clone size, should be integrated into emerging precision medicine frameworks for heart failure.

Several clinically relevant questions merit urgent investigation: Should patients with unexplained HFpEF undergo CHIP testing? Can CHIP status guide selection of anti-inflammatory therapies? Might CHIP screening identify HFpEF patients most likely to benefit from emerging IL-1β inhibitors? Prospective trials addressing these questions are urgently needed and represent a priority for cardiovascular research.

## 4 Molecular mechanisms: the NLRP3-IL-1β axis as the central hub

### 4.1 Epigenetic dysregulation: the origin of hyperinflammation

The most common CHIP mutations affect genes encoding epigenetic regulators—enzymes that modify DNA methylation and chromatin structure without altering the underlying DNA sequence. Understanding how these epigenetic alterations translate into cardiovascular inflammation requires appreciation of their effects on immune cell function, particularly macrophages that play central roles in atherogenesis.

TET2 encodes a methylcytosine dioxygenase that catalyzes the oxidation of 5-methylcytosine to 5-hydroxymethylcytosine, an intermediate in active DNA demethylation. TET2 loss-of-function results in impaired demethylation and, paradoxically, altered methylation patterns at inflammatory gene promoters (1, 6). Specifically, TET2 deficiency leads to hypermethylation of negative regulators of inflammation, thereby releasing the brakes on inflammatory gene expression. This epigenetic “disinhibition” primes macrophages for heightened inflammatory responses.

DNMT3A encodes a *de novo* DNA methyltransferase that establishes new methylation marks. Loss of DNMT3A results in global hypomethylation and aberrant chromatin accessibility, particularly at enhancers and promoters of inflammatory and chemokine genes (37, 38). Single-cell and multi-omic studies have revealed that DNMT3A-deficient macrophages exhibit a hyperinflammatory transcriptional program even prior to stimulation, reflecting epigenetically “primed” chromatin states (39).

A conceptually intriguing question arises—one that reveals the complexity of epigenetic regulation: how do opposite epigenetic perturbations—loss of a demethylase (TET2) vs. loss of a methyltransferase (DNMT3A)—converge on a similar pro-inflammatory phenotype? The answer likely lies in the site-specific nature of epigenetic effects, where both hyper- and hypomethylation at distinct loci can activate inflammatory pathways. Moreover, compensatory changes in histone modifications and transcription factor binding may contribute to phenotypic convergence despite divergent primary mechanisms (40, 41).

### 4.2 The NLRP3 inflammasome: mechanistic epicenter

The NLRP3 inflammasome has emerged as the central molecular effector linking CHIP mutations to cardiovascular inflammation—a finding with direct therapeutic implications. NLRP3 is a cytosolic pattern recognition receptor that, upon activation, assembles into a multiprotein complex facilitating caspase-1 activation and subsequent cleavage of pro-IL-1β into its mature, secreted form.

TET2 deficiency enhances NLRP3 inflammasome function at multiple levels, creating a “perfect storm” for inflammatory activation:

1. Transcriptional priming: TET2-deficient macrophages exhibit increased baseline expression of NLRP3 and pro-IL-1β, lowering the threshold for inflammasome activation (6, 8). This means that even modest inflammatory stimuli can trigger robust IL-1β release.
2. Post-translational activation: Yalcinkaya et al. elucidated a TET2-specific mechanism involving JNK1/BRCC3-mediated deubiquitylation of NLRP3, which promotes inflammasome assembly and activation (42). This pathway appears particularly relevant in the context of cholesterol loading, creating a mechanistic link between dyslipidemia and CHIP-driven inflammation.
3. Synergy with atherogenic stimuli: Cholesterol crystals and oxidized LDL, canonical activators of the NLRP3 inflammasome in atherosclerotic plaques, synergize with TET2 deficiency to amplify IL-1β production (42, 43). This interaction helps explain why CHIP accelerates atherosclerosis specifically in the context of hyperlipidemia.

The functional consequence of NLRP3 hyperactivation is a self-amplifying inflammatory cascade with systemic ramifications. IL-1β secreted by CHIP-mutant macrophages induces IL-6 production, which in turn stimulates hepatic acute-phase reactants including CRP and fibrinogen (1). This systemic inflammatory state promotes endothelial dysfunction, enhances leukocyte adhesion and transmigration, accelerates foam cell formation, and destabilizes atherosclerotic plaques (1, 25).

### 4.3 Beyond NLRP3: alternative and complementary pathways

While the NLRP3-IL-1β axis represents the best-characterized mechanism, emerging evidence suggests additional pathways contribute to CHIP-associated cardiovascular risk—a consideration with important therapeutic implications.

**Type I interferon signaling:** DNMT3A deficiency, in particular, has been linked to upregulation of type I interferon responses, potentially through derepression of endogenous retroelements or mitochondrial DNA sensing (38, 40). Interferons can promote atherosclerosis through mechanisms distinct from IL-1β, suggesting that DNMT3A-CHIP may operate through partially non-overlapping pathways compared to TET2-CHIP.

**Mitochondrial dysfunction.** Pileri and Natoli proposed that CHIP mutations may impair mitochondrial function, leading to release of mitochondrial DNA into the cytosol and activation of cGAS-STING innate immune pathways (44). This mechanism could contribute to inflammation independent of NLRP3.

**Trained immunity.** Epigenetic reprogramming of myeloid progenitors by CHIP mutations may establish a state of “trained immunity,” whereby innate immune cells exhibit heightened and prolonged responsiveness to subsequent stimuli (41). This concept suggests that CHIP effects may persist even after the initial inflammatory trigger is removed—a form of immunological “memory” with pathological consequences.

These alternative mechanisms have important therapeutic implications that we must consider carefully. If CHIP-driven CVD is mediated entirely through NLRP3-IL-1β, then IL-1β inhibition should provide comprehensive protection. However, if parallel pathways contribute, combination therapies or mutation-specific approaches may be required for optimal outcomes. The differential responsiveness of TET2 vs. DNMT3A mutations to anti-inflammatory therapies likely reflects these mechanistic differences.

## 5 Gene-Specific effects: toward a molecular taxonomy of CHIP

### 5.1 TET2: high risk, high reward

Among CHIP driver genes, TET2 mutations consistently confer the highest cardiovascular risk and demonstrate the most robust mechanistic links to inflammation and atherogenesis. This gene-specific pattern has critical implications for both risk stratification and therapeutic intervention.

Epidemiologically, TET2-CHIP is associated with hazard ratios of 1.5–2.5 for incident coronary heart disease and heart failure, exceeding the risk conferred by other common CHIP mutations (18, 35, 45). Mechanistically, TET2 deficiency produces a pronounced NLRP3-dependent inflammatory phenotype, with robust increases in IL-1β and IL-6 secretion that are reversed by inflammasome inhibition (6, 8).

Crucially, TET2-CHIP appears to be the most therapeutically tractable subtype—a finding of paramount clinical importance. Exploratory analyses from the CANTOS trial revealed that patients with TET2-CHIP derived dramatic benefit from canakinumab, with approximately 62% reduction in major adverse cardiovascular events (HR ∼0.38) compared to modest or absent benefit in non-TET2 CHIP carriers (46). Similarly, colchicine appears to preferentially attenuate cardiovascular risk in TET2-mutant individuals (47, 48).

From a clinical standpoint, TET2-CHIP may be conceptualized as an “inflammation-dominant” cardiovascular risk phenotype—one that may be particularly amenable to anti-inflammatory intervention. TET2 mutation status could potentially serve as a companion diagnostic for selecting candidates for IL-1β inhibition or colchicine therapy, though prospective validation remains essential.

### 5.2 DNMT3A: most common, most complex

DNMT3A mutations represent the most prevalent form of CHIP, accounting for approximately 40%–50% of cases (1). Despite this prevalence, the cardiovascular implications of DNMT3A-CHIP are less clear-cut than those of TET2-CHIP—a complexity that clinicians must navigate carefully.

Epidemiological associations between DNMT3A-CHIP and CVD are generally weaker and less consistent across studies (19, 24). Some investigations report null or attenuated associations, leading to debate about whether DNMT3A mutations carry clinically meaningful cardiovascular risk (1, 49).

Mechanistically, DNMT3A-deficient macrophages exhibit a pro-inflammatory phenotype, but the underlying pathways may differ from TET2. Rauch et al. demonstrated that Dnmt3a knockout accelerates atherosclerosis in mice, but the inflammatory profile involves greater upregulation of chemokines and potentially type I interferon responses rather than NLRP3-dominant inflammation (37). Consistent with this, DNMT3A-CHIP appears less responsive to IL-1β inhibition in clinical data (46, 47).

Should DNMT3A-CHIP carriers receive the same intensity of cardiovascular risk management as TET2-CHIP carriers? Based on current evidence, the current evidence argues against equivalent treatment intensity. A more conservative approach for DNMT3A-CHIP, emphasizing traditional risk factor modification while awaiting genotype-specific therapeutic data, seems prudent. However, this recommendation may evolve as our understanding deepens.

### 5.3 Other mutations: a heterogeneous landscape

Beyond TET2 and DNMT3A, several other genes drive clonal hematopoiesis with distinct cardiovascular phenotypes that merit recognition:

**JAK2:** Mutations in JAK2, particularly the V617F variant associated with myeloproliferative neoplasms, confer elevated cardiovascular risk with a unique thrombotic predominance (50). JAK2-mutant cells exhibit enhanced platelet reactivity and neutrophil extracellular trap formation, contributing to arterial and venous thrombosis beyond atherogenesis. Patients with JAK2-CHIP may warrant intensified antithrombotic strategies.

**ASXL1:** ASXL1 mutations appear more strongly associated with hematologic malignancy progression than with cardiovascular outcomes, though limited data exist (19). These patients may require closer hematologic surveillance.

**Splicing factors (SF3B1, SRSF2):** These mutations are more common in the context of myelodysplastic syndromes and carry uncertain cardiovascular implications.

We advocate for the development of a molecular taxonomy that stratifies CHIP by driver gene, clone size, and co-mutation patterns. Such a taxonomy would enable precision approaches to risk assessment and therapy selection—moving beyond the current “one-size-fits-all” approach that fails to capture the biological heterogeneity of CHIP.

## 6 Therapeutic strategies: from proof-of-concept to precision medicine

### 6.1 IL-1β inhibition: lessons from canakinumab

The CANTOS trial (Canakinumab Anti-inflammatory Thrombosis Outcomes Study) was not designed as a CHIP study, yet its retrospective analyses have provided the strongest clinical evidence for mutation-specific cardiovascular therapy—a serendipitous finding with profound implications.

Svensson et al. performed genetic analysis of CANTOS participants and evaluated treatment effects stratified by CHIP status (46). The results were striking and merit emphasis: TET2-CHIP carriers treated with canakinumab experienced a 62% relative risk reduction in major adverse cardiovascular events compared to placebo, whereas non-CHIP carriers showed more modest benefit. This magnitude of effect rivals or exceeds that of any established cardiovascular therapy and suggests that TET2-CHIP identifies a subpopulation with exceptional responsiveness to IL-1β blockade.

Woo et al. extended these findings through proteogenomic analyses, demonstrating that canakinumab reverses inflammatory and erythropoietic abnormalities specifically in CHIP carriers, particularly those with concurrent anemia (51). This suggests that canakinumab may address multiple pathological processes in CHIP patients.

However, critical caveats temper enthusiasm and must be acknowledged honestly. The CHIP analyses were exploratory and retrospective, with modest sample sizes limiting statistical power. Additionally, canakinumab use was associated with increased fatal infections in CANTOS, a risk that may be amplified in elderly patients with comorbidities—the very population most likely to harbor CHIP. The drug's high cost and the absence of FDA approval for cardiovascular indications further limit practical applicability. Nevertheless, these data provide proof-of-concept that mutation-stratified therapy can yield extraordinary benefits.

### 6.2 Colchicine: an accessible alternative

Colchicine has attracted considerable interest as an affordable, widely available anti-inflammatory agent with established cardiovascular benefits—representing a more practical option than canakinumab for most patients. The COLCOT and LoDoCo2 trials demonstrated that low-dose colchicine reduces cardiovascular events in patients with recent myocardial infarction or chronic coronary disease.

Mechanistically, colchicine inhibits microtubule polymerization, impairing NLRP3 inflammasome assembly and IL-1β secretion—precisely the pathway hyperactivated in TET2-CHIP (52). Preclinical studies by Zuriaga et al. demonstrated that colchicine prevents accelerated atherosclerosis in Tet2-mutant mice, providing mechanistic support for mutation-specific efficacy (47). Human biobank analyses confirmed that colchicine use attenuates the association between TET2-CHIP and myocardial infarction, whereas no such attenuation was observed for DNMT3A-CHIP (47, 48).

From a practical standpoint, colchicine offers significant advantages: it is inexpensive, orally administered, and already approved for cardiovascular use. Whether TET2-CHIP status should influence colchicine prescribing—favoring its use in mutation carriers—is a provocative question that merits serious consideration. While prospective data are lacking, the convergent mechanistic and observational evidence provides a reasonable basis for considering colchicine as a preferred anti-inflammatory agent in TET2-CHIP carriers with atherosclerotic CVD.

### 6.3 IL-6 pathway inhibition

IL-6, produced downstream of IL-1β, amplifies the inflammatory cascade and stimulates hepatic acute-phase responses. Genetic studies have demonstrated that inherited variants reducing IL-6 signaling attenuate cardiovascular risk in CHIP carriers (53), providing a rationale for therapeutic IL-6 blockade.

Liu et al. showed that IL-6 receptor blockade alleviates atherosclerosis in Tet2-deficient mice, supporting the therapeutic potential of this approach (54). Tocilizumab, an IL-6 receptor antagonist approved for rheumatoid arthritis, could potentially be repurposed for CHIP-associated CVD, though cardiovascular safety data from rheumatologic populations have been mixed and warrant careful evaluation.

### 6.4 Emerging targets and future therapies

Several novel therapeutic approaches are under investigation, representing the next frontier in CHIP-targeted therapy:

**Selective NLRP3 inhibitors:** Small-molecule NLRP3 inhibitors (e.g., MCC950, OLT1177) offer targeted inflammasome blockade without broad immunosuppression. These agents are in early clinical development for inflammatory diseases and could represent ideal candidates for CHIP-associated CVD, potentially offering better safety profiles than systemic cytokine inhibition.

**Epigenetic modulators:** Theoretically, drugs that restore normal epigenetic patterns might reverse the proinflammatory programming of CHIP-mutant cells. However, the therapeutic window for such agents remains uncertain, and off-target effects are a significant concern.

**Clonal targeting:** An intriguing frontier is the selective elimination or suppression of CHIP clones themselves—essentially treating the upstream cause rather than downstream consequences. Approaches under exploration include targeted cytotoxic agents, immunotherapy, and gene editing, though all remain far from clinical application.

### 6.5 A framework for clinical decision-making

How should the practicing cardiologist incorporate CHIP into patient management today? Based on current evidence, a provisional clinical framework may be considered:

1. **Current guidance**: Routine CHIP screening is not recommended in the general population, as evidence-based management algorithms are lacking and the clinical utility remains unproven.
2. **Incidental CHIP findings**: When CHIP is discovered incidentally (e.g., through tumor genomic profiling or research studies), mutation type and VAF should be documented. TET2 mutations with VAF >10% warrant heightened cardiovascular vigilance and consideration of enhanced risk factor management.
3. **Risk factor intensification**: All CHIP carriers, regardless of mutation, should receive aggressive management of traditional cardiovascular risk factors. There is no justification for therapeutic nihilism.
4. **Anti-inflammatory therapy**: For TET2-CHIP carriers with established atherosclerotic CVD, colchicine may be considered preferentially over other secondary prevention options, given its favorable efficacy-safety profile and the mechanistic rationale. Canakinumab, if available and affordable, represents a rational choice in high-risk TET2-CHIP patients who have failed or cannot tolerate other therapies.
5. **Hematologic monitoring**: Annual complete blood counts are reasonable to monitor for progression toward hematologic malignancy, which occurs in approximately 0.5%–1% of CHIP carriers annually.

## 7 Research gaps and future directions

### 7.1 Urgent unanswered questions

Despite rapid progress, critical gaps impede clinical translation and must be addressed:

**Prospective, genotype-stratified trials:** The field's most pressing need is prospective clinical trials testing anti-inflammatory therapies specifically in CHIP carriers, stratified by mutation type. Such trials would definitively establish whether the retrospective associations from CANTOS translate into actionable clinical benefit. Until such data exist, our therapeutic recommendations remain extrapolations rather than evidence-based guidelines.

**Non-European populations:** The overwhelming majority of CHIP data derive from European-ancestry cohorts—a significant limitation that raises concerns about health equity. CHIP prevalence, mutation spectrum, and cardiovascular associations may differ across racial and ethnic groups, with important implications for global applicability of CHIP-targeted therapies.

**Natural history studies:** The dynamics of CHIP clone expansion—including factors promoting or restraining growth—remain poorly understood. Longitudinal studies tracking VAF trajectories alongside cardiovascular outcomes would inform optimal monitoring intervals and intervention thresholds. Currently, we lack guidance on whether clone growth rate predicts cardiovascular events.

### 7.2 Technological opportunities

Emerging technologies offer new avenues for CHIP research and clinical application:

**Liquid biopsy and myeloid cell enrichment strategies:** While circulating cell-free DNA (cfDNA) analysis has been proposed for CHIP monitoring, important limitations merit recognition. cfDNA sequencing is not substantially less invasive than peripheral blood leukocyte sequencing—both require phlebotomy—and cfDNA may have lower sensitivity for detecting CHIP at low variant allele fractions compared with direct sequencing of white blood cells. A more promising approach may be targeted enrichment of circulating myeloid cells prior to sequencing, which could improve sensitivity and specificity while maintaining the practicality of peripheral blood sampling and facilitating serial CHIP monitoring.

**Single-cell multi-omics:** Single-cell ATAC-seq and chromatin accessibility profiling are revealing the epigenetic heterogeneity of CHIP clones and their immune microenvironment, potentially identifying new therapeutic targets and biomarkers. It should be noted that single-cell RNA sequencing alone is not an optimal method for identifying the somatic DNA variants that define CHIP clones; DNA-based next-generation sequencing remains the gold standard for detecting clonal mutations at the variant allele fractions relevant to CHIP detection and monitoring.

**Artificial intelligence:** Machine learning algorithms integrating CHIP genotype with traditional risk factors, imaging data, and biomarkers could generate superior cardiovascular risk prediction models, enabling more precise patient stratification.

### 7.3 Toward a new subspecialty: cardio-hematology

CHIP research exemplifies the convergence of cardiovascular medicine and hematology in ways that neither field anticipated. We propose the emergence of “Cardio-Hematology” as a subspecialty dedicated to the intersection of clonal hematopoiesis, hematologic malignancies, and cardiovascular disease. Such a field would integrate expertise in genomics, inflammation biology, and clinical cardiology to optimize care for the growing population of patients at this crossroads.

Establishing dedicated Cardio-Hematology clinics—analogous to the successful Cardio-Oncology model—could facilitate coordinated care, prospective registries, and translational research. Several tertiary medical centers have already developed dedicated CHIP clinics for multidisciplinary management and counseling of patients with clonal hematopoiesis (55), demonstrating the clinical feasibility and value of this model. The infrastructure developed for Cardio-Oncology over the past decade provides a further template for broader subspecialty development.

An important and underrecognized dimension of the emerging Cardio-Hematology subspecialty is the shared biological risk for cardiovascular disease and cancer mediated by CHIP. Cardiovascular disease is highly prevalent among cancer survivors, and growing evidence suggests that CVD may in turn increase cancer risk (56, 57). Critically, CHIP has been specifically associated with increased cardiovascular disease risk in patients with solid tumors receiving cardiotoxic cancer therapies (58), identifying clonal hematopoiesis as a potential mechanistic mediator of treatment-related cardiotoxicity. This convergence of cardiovascular and oncologic risk argues for comprehensive, multidisciplinary care frameworks—incorporating hematology, cardiology, and oncology—to address the full spectrum of CHIP-related morbidity.

## 8 Conclusions

Clonal hematopoiesis of indeterminate potential has substantially advanced our understanding of cardiovascular pathogenesis by demonstrating how somatic mutations in hematopoietic stem cells amplify the bone marrow–heart axis—a relationship whose biological foundations were established by prior work in hematopoietic and vascular biology—to accelerate atherosclerosis, heart failure, and cardiovascular mortality (59–60). Current evidence supports CHIP not merely as a biomarker of biological aging but as a causal and potentially modifiable driver of cardiovascular inflammation.

Several key messages emerge from this review:

1. **CHIP is an independent cardiovascular risk factor**, conferring a 1.5- to 2-fold increased risk of coronary heart disease and mortality, comparable in magnitude to established risk factors such as diabetes. This recognition demands integration of CHIP into our conceptual framework of cardiovascular risk.
2. **Gene-specific heterogeneity is fundamental** and must guide clinical decision-making. TET2 mutations confer the highest cardiovascular risk and demonstrate the greatest responsiveness to anti-inflammatory therapy. DNMT3A mutations are more common but show weaker and less consistent cardiovascular associations (61).
3. **The NLRP3-IL-1β axis is the mechanistic epicenter** of CHIP-driven cardiovascular inflammation, particularly for TET2-CHIP. This pathway represents a validated therapeutic target with multiple druggable nodes.
4. **Precision medicine approaches are within reach**. Mutation-stratified therapy—using IL-1β inhibitors or colchicine preferentially in TET2-CHIP carriers—represents a tangible near-term opportunity for clinical translation that could be implemented with current tools.
5. **Critical gaps remain**, including the absence of prospective, genotype-stratified clinical trials and limited data in non-European populations. Addressing these gaps is essential for the field to advance responsibly.

As cardiovascular medicine evolves from a disease-centered to a mechanism-centered discipline, CHIP exemplifies the potential of precision approaches to transform patient care. The next decade will determine whether this potential is realized—whether CHIP testing becomes routine in cardiovascular risk assessment, and whether mutation-guided therapy becomes a pillar of preventive cardiology.

The bone marrow-heart axis is open for investigation. The journey from discovery to clinical impact is underway, and the destination—personalized cardiovascular medicine guided by somatic genetics—is now visible on the horizon.

## References

[1] Jaiswal S Libby P. Clonal haematopoiesis: connecting ageing and inflammation in cardiovascular disease. Nat Rev Cardiol. (2020) 17(3):137–44. 10.1038/s41569-019-0247-5 31406340 PMC9448847

[2] Yvan-Charvet L Pagler T Gautier EL Avagyan S Siry RL Han S ATP-binding cassette transporters and HDL suppress hematopoietic stem cell proliferation. Science. (2010) 328(5986):1689–93. 10.1126/science.1189731 20488992 PMC3032591

[3] Libby P Sidlow R Lin AE Gupta D Jones LW Moslehi J Clonal hematopoiesis: crossroads of aging, cardiovascular disease, and cancer. J Am Coll Cardiol. (2019) 74(4):567–77. 10.1016/j.jacc.2019.06.007 31345432 PMC6681657

[4] Steensma DP Bejar R Jaiswal S Lindsley RC Sekeres MA Hasserjian RP Clonal hematopoiesis of indeterminate potential and its distinction from myelodysplastic syndromes. Blood. (2015) 126(1):9–16. 10.1182/blood-2015-03-631747 25931582 PMC4624443

[5] Khetarpal SA Qamar A Bick AG Fuster JJ Kathiresan S Jaiswal S Clonal hematopoiesis of indeterminate potential reshapes age-related CVD. J Am Coll Cardiol. (2019) 74(4):578–86. 10.1016/j.jacc.2019.05.045 31345433 PMC6662618

[6] Fuster JJ MacLauchlan S Zuriaga MA Polackal MN Ostriker AC Chakraborty R Clonal hematopoiesis associated with TET2 deficiency accelerates atherosclerosis development in mice. Science. (2017) 355(6327):842–7. 10.1126/science.aag1381 28104796 PMC5542057

[7] Jaiswal S Natarajan P Silver AJ Gibson CJ Bick AG Shvartz E Clonal hematopoiesis and risk of atherosclerotic cardiovascular disease. N Engl J Med. (2017) 377(2):111–21. 10.1056/NEJMoa1701719 28636844 PMC6717509

[8] Sano S Oshima K Wang Y MacLauchlan S Katanasaka Y Zuriaga MA Tet2-Mediated clonal hematopoiesis accelerates heart failure through a mechanism involving the IL-1β/NLRP3 inflammasome. J Am Coll Cardiol. (2018) 71(8):875–86. 10.1016/j.jacc.2017.12.037 29471939 PMC5828038

[9] Jaiswal S Fontanillas P Flannick J Manning A Grauman PV Mar BG Age-related clonal hematopoiesis associated with adverse outcomes. N Engl J Med. (2014) 371(26):2488–98. 10.1056/NEJMoa1408617 25426837 PMC4306669

[10] Yang SJ Kim N Park HK Lee JS Cho KH Kim HJ Clonal hematopoiesis and risk of ischemic stroke: a hospital-based cohort study. Stroke. (2018) 49(10):2507–10. 10.1161/STROKEAHA.118.022374

[11] Bhattacharya R Zekavat SM Obermeier P Haessler J Fornage M Raffield L Clonal hematopoiesis is associated with higher risk of stroke. Stroke. (2022) 53(3):788–97. 10.1161/STROKEAHA.121.037388 34743536 PMC8885769

[12] Schuermans A Weng LC Khurshid S Chaffin MD Perz S van der Heijden JF Clonal hematopoiesis of indeterminate potential and atrial fibrillation. Eur Heart J. (2024) 45(32):2935–48. 10.1093/eurheartj/ehae432

[13] Ahn JH Seo MH Hwang J Kim K Park JH Yoon JA Clonal haematopoiesis of indeterminate potential and risk of atrial fibrillation. Eur Heart J. (2024) 45(12):1039–50. 10.1093/eurheartj/ehad829

[14] Schuermans A Cho SMJ Uddin MM Darke JA Rosenberg Y Sahoo PK Clonal hematopoiesis and incident myocarditis and pericarditis. JAMA Cardiol. (2025) 10(1):88–92. 10.1001/jamacardio.2024.3991

[15] Zekavat SM Vaitinadin NS Bhattacharya R Uddin MM Pirruccello JP Griffin GK Clonal hematopoiesis of indeterminate potential is associated with peripheral artery disease. Nat Cardiovasc Res. (2023) 2(11):1154–64. 10.1038/s44161-023-00350-7

[16] Nakao T Bick AG Taub MA Pirruccello JP Griffin GK Natarajan P Clonal hematopoiesis of indeterminate potential and aortic valve stenosis. J Am Coll Cardiol. (2023) 81(14):1329–41. 10.1016/j.jacc.2023.01.040

[17] Rhee JW Bolton KL Gupta D Weeks LD Bick AG Tall AR Clonal hematopoiesis and its cardiovascular implications: a scientific statement from the American Heart Association. Circulation. (2026) 153(11):e940–e952. 10.1161/CIR.0000000000001272 41664921

[18] Gumuser E Schuermans A Cho SMJ Sporn ZA Uddin MM Paruchuri K Clonal hematopoiesis of indeterminate potential predicts adverse outcomes in patients with atherosclerotic cardiovascular disease. J Am Coll Cardiol. (2023) 81(20):1996–2009. 10.1016/j.jacc.2023.03.401 37197843 PMC10249057

[19] Singh J Li N Ashrafi E Thao LTP Curtis DJ Wood EM Clonal hematopoiesis of indeterminate potential as a prognostic factor: a systematic review and meta-analysis. Blood Adv. (2024) 8(15):3771–84. 10.1182/bloodadvances.2024013228 38838228 PMC11298876

[20] McQuilten Z Thao L Bick A Byars S Chan AT Ford L Clonal hematopoiesis and cardiovascular disease and bleeding risk and the effectiveness of aspirin. JAMA Cardiol. (2025) 10(12):1257. 10.1001/jamacardio.2025.3756 41091476 PMC12529328

[21] Assmus B Cremer S Kirschbaum K Culmann D Kiefer K Dorsheimer L Clonal haematopoiesis in chronic ischaemic heart failure: prognostic role of clone size for DNMT3A- and TET2-driver gene mutations. Eur Heart J. (2021) 42(3):257–65. 10.1093/eurheartj/ehaa845 33241418

[22] Karakasis P Lefkou E Pamporis K Farmakis D Patoulias D Antoniadis AP Association of clonal haematopoiesis with heart failure incidence and outcomes: a systematic review and meta-analysis. Eur J Heart Fail. (2025) 27(9):1775–85. 10.1002/ejhf.3637 40064512 PMC12502460

[23] Bhattacharya R Bick A. Clonal hematopoiesis of indeterminate potential: an expanding genetic cause of cardiovascular disease. Curr Atheroscler Rep. (2021) 23(12):66. 10.1007/s11883-021-00966-9 34468876 PMC8543762

[24] Pan Y Vlasschaert C Rao V Akwo EA Hixson JE Uddin MM Association of clonal hematopoiesis of indeterminate potential with cardiovascular events in patients with CKD. J Am Soc Nephrol. (2025) 36(9):1775–85. 10.1681/asn.0000000671 40202812 PMC12416944

[25] Von Scheidt M Adkar SS Krefting J Hoermann G Meggendorfer M Bauer S Clonal haematopoiesis of indeterminate potential and mortality in coronary artery disease. Eur Heart J. (2025) 46(21):1892–904. 10.1093/eurheartj/ehaf602 PMC12831187 40900105

[26] Simitsis P Nohria A Kelleher J Boulet J Wanderley MRB Natarajan P Clonal hematopoiesis of indeterminate potential and long-term outcomes in heart transplantation. J Card Fail. (2024) 30(10):1234–41. 10.1016/j.cardfail.2024.05.011 38885783

[27] Muller M Liu R Hu J Held C Kullo I McManus B Abstract 4124065: clonal hematopoiesis of indeterminate potential (CHIP) in chronic coronary artery disease: a report from the ISCHEMIA trials biorepository. Circulation. (2024) 150(Suppl 1):4124065. 10.1161/circ.150.suppl_1.4124065 PMC12403068 40207358

[28] Ezzat D Uddin M Xue L Pershad Y Zhang S Collins JM Clonal hematopoiesis and cardiovascular outcomes in older women. J Am Coll Cardiol. (2025) 86(12):1093–106. 10.1016/j.jacc.2025.07.058 40864016 PMC12557340

[29] Shi C Aboumsallem J Suthahar N de Graaf AO Jansen JH van Zeventer IA Clonal haematopoiesis of indeterminate potential: associations with heart failure incidence, clinical parameters and biomarkers. Eur J Heart Fail. (2023) 25(1):4–13. 10.1002/ejhf.2715 36221810 PMC10092539

[30] Flynn S Schuermans A Uddin MM Darke JA Cho SMJ Rosenberg Y Clonal hematopoiesis and incident heart failure. JAMA Cardiol. (2026) 11(2):126–35. 10.1001/jamacardio.2025.4028 41206888 PMC12598579

[31] Dorsheimer L Assmus B Rasper T Ortmann CA Ecke A Abou-El-Ardat K Association of mutations contributing to clonal hematopoiesis with prognosis in chronic ischemic heart failure. JAMA Cardiol. (2019) 4(1):32–40. 10.1001/jamacardio.2018.3965 PMC6439691 30566180

[32] Pascual-Figal D Bayés-Genís A Díez-Díez M Bayes-Genis A Hernández-Vicente Á Vázquez-Andrés D Clonal hematopoiesis and risk of progression of heart failure with reduced left ventricular ejection fraction. J Am Coll Cardiol. (2021) 77(14):1747–59. 10.1016/j.jacc.2021.02.028 33832602

[33] Schiattarella GG Altamirano F Tong D French KM Villalobos E Kim SY Nitrosative stress drives heart failure with preserved ejection fraction. Nature. (2019) 568(7752):351–6. 10.1038/s41586-019-1100-z 30971818 PMC6635957

[34] Cochran JD Yura Y Thel MC Doviak H Polizio AH Arai Y Clonal hematopoiesis in clinical and experimental heart failure with preserved ejection fraction. Circulation. (2023) 148(15):1165–78. 10.1161/circulationaha.123.064170 37681311 PMC10575571

[35] Schuermans A Honigberg M Raffield L Uddin MM Cho SMJ Darke JA Clonal hematopoiesis and incident heart failure with preserved ejection fraction. JAMA Netw Open. (2024) 7(1):e2353244. 10.1001/jamanetworkopen.2023.53244 38270950 PMC10811556

[36] Pandey A Thomas T Ji Y Kroger B Irion C Kalkan F Abstract 4144372: tET2-mutant clonal hematopoiesis promotes heart failure with preserved ejection fraction. Circulation. (2024) 150(Suppl 1):4144372. 10.1161/circ.150.suppl_1.4144372 PMC12805981 40455840

[37] Rauch PJ Silver AJ Gopakumar J McConkey M Sinha E Fefer M Loss-of-Function mutations in Dnmt3a and Tet2 lead to accelerated atherosclerosis and convergent macrophage phenotypes in mice. Blood. (2018) 132(Suppl 1):118288. 10.1182/blood-2018-99-118288

[38] Rodrigues K Gopakumar J Weng Z Mitchell SR Maurer M Nachun D Multi-omic profiling of macrophages lacking Tet2 or Dnmt3a reveals mechanisms of hyper-inflammation in clonal hematopoiesis. Blood. (2023) 142(Suppl 1):187890. 10.1182/blood-2023-187890

[39] Abplanalp WT Cremer S John D Hoffmann J Schuhmacher B Merten M Clonal hematopoiesis–driver DNMT3A mutations Alter immune cells in heart failure. Circ Res. (2021) 128(2):216–28. 10.1161/circresaha.120.317104 33155517

[40] Cobo I Tanaka T Mangalhara KC Chandra Mangalhara K Lana A Yeang C DNA methyltransferase 3 alpha and TET methylcytosine dioxygenase 2 restrain mitochondrial DNA-mediated interferon signaling in macrophages. Immunity. (2022) 55(8):1386–401. 10.1016/j.immuni.2022.06.022 35931086 PMC9718507

[41] Belizaire R Wong W Robinette M Ebert B. Clonal haematopoiesis and dysregulation of the immune system. Nat Rev Immunol. (2023) 23(9):595–610. 10.1038/s41577-023-00843-3 36941354 PMC11140722

[42] Yalcinkaya M Liu W Thomas LA Olszewska M Xiao T Abramowicz S BRCC3-Mediated NLRP3 deubiquitylation promotes inflammasome activation and atherosclerosis in Tet2 clonal hematopoiesis. Circulation. (2023) 148(22):1764–77. 10.1161/circulationaha.123.065344 37781816 PMC10872582

[43] Yalcinkaya M Liu W Xiao T Abramowicz S Wang N Westerterp M Abstract 108: inflammasome activation via BRCC3-mediated NLRP3 deubiquitylation promotes atherosclerosis in Tet2 clonal hematopoiesis. Arterioscler Thromb Vasc Biol. (2024) 44(Suppl 1):108. 10.1161/atvb.44.suppl_1.10837942609

[44] Pileri F Natoli G. Clonal hematopoiesis, inflammation, and cardiovascular disorders: a mitochondrial connection. Trends Immunol. (2022) 43(11):865–7. 10.1016/j.it.2022.07.009 35945112

[45] Honigberg MC Reiner A Roberts M Kooperberg C Desai P Bick AG Abstract 15818: association of clonal hematopoiesis of indeterminate potential with incident heart failure with preserved ejection fraction. Circulation. (2023) 148(Suppl 1):15818. 10.1161/circ.148.suppl_1.15818

[46] Svensson EC Madar A Campbell CD Sultan M Healey ML Xu H TET2-driven clonal hematopoiesis and response to canakinumab: an exploratory analysis of the CANTOS randomized clinical trial. JAMA Cardiol. (2022) 7(5):521–8. 10.1001/jamacardio.2022.0386 35385050 PMC8988022

[47] Zuriaga MA Yu Z Matesanz N Truong B Ramos-Neble BL Asensio-López MC Colchicine prevents accelerated atherosclerosis in TET2-mutant clonal haematopoiesis. Eur Heart J. (2024) 45(46):4601–15. 10.1093/eurheartj/ehae546 39212933 PMC11560281

[48] Mohammadnia N Xue L Vestjens L Uddin MM Niroula A de Kleijn DPV Colchicine and longitudinal dynamics of clonal hematopoiesis. J Am Coll Cardiol. (2025) 86(21):1983–96. 10.1016/j.jacc.2025.08.025 40892620 PMC12557339

[49] Mooney L Goodyear CS Chandra T Kirschner K Copland M Petrie MC Clonal haematopoiesis of indeterminate potential: intersections between inflammation, vascular disease and heart failure. Clin Sci. (2021) 135(7):991–1007. 10.1042/cs20200306 PMC8055963 33861346

[50] Leiva O Hobbs G Ravid K Libby P. Cardiovascular disease in myeloproliferative neoplasms. JACC CardioOncol. (2022) 4(2):166–82. 10.1016/j.jaccao.2022.04.002 35818539 PMC9270630

[51] Woo J Lu D Lewandowski A Xu H Serrano P Healey M Effects of IL-1β inhibition on anemia and clonal hematopoiesis in the randomized CANTOS trial. Blood Adv. (2023) 7(24):7471–84. 10.1182/bloodadvances.2023011578 37934948 PMC10758744

[52] Todorovski A Wang T Carrier M Bhatt DL Xu Y. CHIP away at the marrow-clot connection: inflammation, clonal hematopoiesis, and thromboembolic disease. Blood Adv. (2025) 9(2):343–53. 10.1182/bloodadvances.2024014430 39561373 PMC11787476

[53] Bick AG Pirruccello JP Griffin GK Gupta N Gabriel S Saleheen D Genetic interleukin 6 signaling deficiency attenuates cardiovascular risk in clonal hematopoiesis. Circulation. (2020) 141(2):124–31. 10.1161/circulationaha.119.044362 31707836 PMC7008855

[54] Liu W Yalcinkaya M Maestre IF Olszewska M Ampomah PB Heimlich JB Blockade of IL-6 signaling alleviates atherosclerosis in Tet2-deficient clonal hematopoiesis. Nat Cardiovasc Res. (2023) 2(6):572–86. 10.1038/s44161-023-00281-3 37539077 PMC10399458

[55] Bolton KL Ptashkin RN Gao T Zehir A Patel M Gupta D Clonal hematopoiesis and cardio-hematology. Hematol Oncol Clin North Am. (2020) 34(2):357–67. 10.1016/j.hoc.2019.11.006 32089215

[56] Peterson LR Brown SA Dent S Barac A Moslehi J Lenihan DJ Cardiotoxicity and its prevention in cancer therapy. JACC CardioOncol. (2022) 4(3):336–9. 10.1016/j.jaccao.2022.07.003

[57] Evans MA Chang A Libby P Bick AG Natarajan P Moslehi J CHIP as a shared risk factor for cardiovascular disease and cancer. JACC CardioOncol. (2025) 7(1):1–10. 10.1016/j.jaccao.2025.01.00139896126

[58] Shyr DC Bhatt DL Bhattacharya R Zekavat SM Uddin MM Moslehi J Clonal hematopoiesis of indeterminate potential and cardiovascular disease risk after cardiotoxic cancer therapy. JAMA Oncol. (2026) 12(3):e255678. 10.1001/jamaoncol.2025.5678 PMC12784258 41505144

[59] Oren O Small AM Libby P. Clonal hematopoiesis and atherosclerosis. J Clin Invest. (2024) 134(8):e180066. 10.1172/jci180066 39352379 PMC11444192

[60] Sikking M Stroeks S Waring O Henkens MTHM Riksen NP Hoischen A Clonal hematopoiesis of indeterminate potential from a heart failure specialist’s point of view. J Am Heart Assoc. (2023) 12(10):e030603. 10.1161/jaha.123.030603 37489738 PMC10492961

[61] Wang Y Sano S Yura Y Ke Z Oshima K Ogawa H Tet2-mediated clonal hematopoiesis in non-conditioned mice accelerates age-associated cardiac dysfunction. JCI Insight. (2020) 5(6):e135204. 10.1172/jci.insight.135204 32154790 PMC7213793

